# Ivermectin attenuates nicotine-induced reward-like behaviors in mice

**DOI:** 10.17305/bb.2025.13026

**Published:** 2025-09-26

**Authors:** Mustafa Enes Demirel, Abdurrahman Ekici, Oruç Yunusoğlu

**Affiliations:** 1 Department of Emergency Medicine, Bolu Abant Izzet Baysal University, Faculty of Medicine, Bolu, Türkiye; 2 Department of Parasitology, Yuzuncu Yıl University, Faculty of Medicine, Van, Türkiye; 3 Department of Pharmacology, Bolu Abant Izzet Baysal University, Faculty of Medicine, Bolu, Türkiye

**Keywords:** Ivermectin, nicotine, CPP, emergency medicine, health burden, dependence

## Abstract

Nicotine addiction poses a significant public health threat, particularly within the realm of emergency medicine, where it is associated with serious complications, including cardiovascular events and respiratory distress. The limited effectiveness of current pharmacological treatments for nicotine dependence underscores the urgent need for innovative and effective therapeutic approaches. Recent studies have shown that ivermectin, an antiparasitic agent, modulates the GABAergic, glutamatergic, and purinergic systems, which are implicated in the pathophysiology of addiction. This study aimed to examine the effects of ivermectin on the acquisition, extinction, and reinstatement of nicotine dependence in mice, utilizing the conditioned place preference (CPP) test, a widely recognized methodology in drug addiction research. Ivermectin (1 and 5 mg/kg, i.p.) was co-administered with nicotine (0.5 mg/kg, i.p.) over three consecutive days during the acquisition phase of nicotine dependence. In a separate experiment, the influence of ivermectin on the reinstatement of nicotine-induced CPP was assessed following an extinction period, using a single nicotine priming injection (0.1 mg/kg). Results indicated that ivermectin (1 and 5 mg/kg) significantly reduced the development of nicotine dependence (*P* < 0.05). Furthermore, ivermectin (5 mg/kg) facilitated the extinction of nicotine-induced CPP (*P* < 0.01) and attenuated the reinstatement of nicotine-induced CPP triggered by a priming dose of nicotine (*P* < 0.01). In contrast, administration of the lower dose of ivermectin (1 mg/kg) did not yield statistically significant effects on either the extinction or reinstatement phases (*P* > 0.05). Additionally, nicotine administration, alone or in combination with ivermectin at the tested doses, did not produce significant changes in motor coordination or locomotor activity. These findings suggest that ivermectin may attenuate both the acquisition and reinstatement of nicotine-induced CPP while facilitating the extinction of nicotine dependence. Collectively, the results indicate that ivermectin holds potential as a therapeutic agent in the treatment of nicotine addiction.

## Introduction

Nicotine addiction is one of the most prevalent and preventable causes of morbidity and mortality in emergency departments worldwide. Both acute and chronic smoking-related complications place a significant burden on public health systems and emergency care services. Research indicates that approximately 5% of adult emergency visits, 6.8% of hospital admissions from emergency departments, and 10% of total hospital costs are attributable to smoking-related conditions [1, 2]. These statistics underscore the prevalence of tobacco-related health issues, particularly within emergency departments [2].

As the primary psychoactive component of tobacco, nicotine rapidly induces dependence by modulating central nervous system (CNS) pathways [3–5]. It achieves this by binding to nicotinic acetylcholine receptors, which stimulates the release of various neurotransmitters. This neurochemical activity is fundamental to the diverse physiological and behavioral effects experienced by tobacco users. Notably, neurotransmitters such as dopamine, glutamate, and GABA play critical roles in the neuroadaptations associated with nicotine dependence and in the withdrawal symptoms that arise upon cessation [4, 5].

Withdrawal symptoms such as agitation, anxiety, and tachycardia frequently occur in emergency departments following nicotine cessation, presenting additional challenges for patient management. Furthermore, acute exacerbations related to smoking—such as chronic obstructive pulmonary disease (COPD), asthma, myocardial infarction, stroke, and acute cerebral ischemia—significantly impact the workload of emergency departments [6–11]. Therefore, research into innovative pharmacological interventions for nicotine addiction is a critical priority in emergency medicine, addressing both preventive healthcare and the management of acute complications.

Current pharmacotherapies for nicotine addiction include nicotine replacement therapy (NRT), varenicline, and bupropion, which alleviate withdrawal symptoms and nicotine cravings by modulating dopaminergic, noradrenergic, and nicotinic pathways [12, 13]. Although these agents enhance short-term cessation rates, achieving long-term abstinence remains challenging; over 60% of individuals relapse within one year [14].

Ivermectin, an FDA-approved antiparasitic agent, functions as a positive allosteric modulator of GABA-A and P2X4 receptors [15–17]. Experimental studies indicate that ivermectin can reduce alcohol consumption and may serve as a potential treatment for alcohol use disorder [18–21]. By enhancing GABAergic inhibition and regulating P2X4-mediated signaling, ivermectin may help restore the excitatory-inhibitory imbalance associated with nicotine dependence [15–17, 21, 22]. Furthermore, it has been reported that ivermectin influences the cholinergic system in reward centers, while nicotine modifies purinergic signaling within these regions [16, 23]. During nicotine withdrawal, the activation of P2X and P2Y1 receptors increases the firing activity of cholinergic neurons, whereas the administration of nicotine with P2X agonists enhances synaptic responses [23–28]. These findings suggest that the purinergic system could be a viable therapeutic target in the development of interventions for addiction, withdrawal, and relapse phases.

The conditioned place preference (CPP) paradigm is utilized to evaluate the associative rewarding properties of various stimuli, including pharmacological agents, social engagement, palatable food, and sexual behavior [29–31]. This model has successfully investigated the addictive potential of substances such as morphine, fentanyl, benzodiazepines, alcohol, amphetamines, and nicotine [30–32]. Notably, numerous studies have demonstrated that nicotine induces a contextual preference within CPP paradigms. Additionally, recent adaptations of the CPP model have been applied to human populations to explore approach-avoidance responses to addictive stimuli and how these responses vary based on contextual factors [30, 33, 34].

The limited effectiveness of existing treatments underscores the need for alternative pharmacological strategies that target broader neurobiological mechanisms, including glutamatergic, GABAergic, and purinergic pathways. The literature suggests that ivermectin may modulate these systems implicated in nicotine addiction, prompting further investigation into its potential therapeutic effects.

## Materials and methods

All experimental procedures adhered to the ethical guidelines established by the local animal ethics committee. The care and handling of animals, along with all interventions, were conducted in accordance with the principles set forth in the Universal Declaration of Animal Rights. Male Swiss albino mice (8 weeks old, weighing 22–25 g) were maintained under standardized laboratory conditions, which included controlled humidity (50%–70%), ambient temperature (19 ± 2 ^∘^C), and a 12-h light/dark cycle. Throughout the study, the animals had unrestricted access to standard rodent chow and water.

### Drugs

Nicotine hydrogen tartrate was dissolved in sterile physiological saline (0.9% NaCl) immediately prior to administration. The pH of the nicotine solutions was adjusted to 7.4 using dilute 0.1 M NaOH. The nicotine doses are expressed as the free base. All drugs were sourced from Sigma Chemicals (St. Louis, MO, USA) and administered intraperitoneally (i.p.) at a volume of 10 mL/kg. Ivermectin was dissolved in 1% DMSO, while normal saline (0.9% NaCl) served as the control. All injections were performed at room temperature.

### CPP apparatus

**Figure 1.**
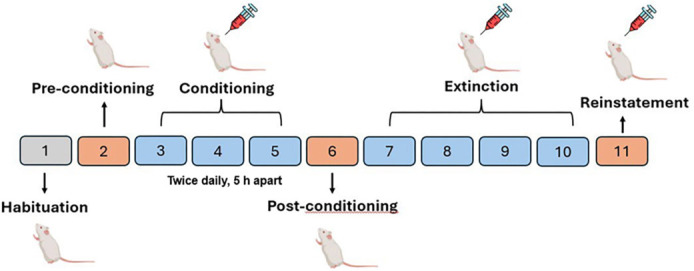
**Timeline and dosing scheme for the CPP paradigm.** The CPP procedure consisted of habituation (Day 1), preconditioning test (Day 2), conditioning (Days 3–5), post-conditioning test (Day 6), extinction (Days 7–10), and reinstatement (Day 11). During habituation and test sessions, mice freely explored both chambers of the apparatus, while during conditioning, they were confined to one chamber following drug or vehicle administration (twice daily, 5 h apart). Extinction and reinstatement were assessed under the same conditions, with appropriate pretreatments (vehicle, nicotine, or ivermectin) administered as indicated. Abbreviations: CPP: Conditioned place preference.

The CPP apparatus comprised two conditioning chambers of identical dimensions (20 × 20 × 20 cm): one featured white vertical lines on the walls with a grid floor, while the other showcased black horizontal lines on the walls with a perforated floor. These main chambers were interconnected by sliding doors, facilitating access between them. The CPP protocol was adapted from McKendrick and Graziane [35] with minor modifications and included the following phases: habituation, preconditioning test, conditioning, post-conditioning test, extinction, and nicotine re-exposure test. During the test sessions, a webcam recorded the duration of time spent in each chamber. Drug-induced CPP acquisition was assessed by comparing the time spent in the nicotine-paired chamber to that in the vehicle (VEC)-paired chamber. Subsequently, the recorded videos were analyzed by a blinded observer to ensure an unbiased evaluation. Each phase was conducted as detailed below (see Figure 1):

**Habituation phase (Day 1):** Mice were placed in the center of the CPP apparatus with the doors open, allowing unrestricted access to both chambers for 5 min. No injections were administered during this phase.

**Pre-conditioning test (Day 2):** To establish baseline chamber preference, mice were again positioned in the center of the apparatus with the doors open, permitting free access to both compartments for 15 min. No injections were given on this day.

**Effect of conditioning and ivermectin on the acquisition of nicotine dependence (Days 3–5):** A neutral design was employed, as mice exhibited no preference for either chamber during the pre-conditioning test. Therefore, conditioning was conducted in a counterbalanced manner. Over three consecutive days, with the doors closed, animals were confined to one of the conditioning chambers. In the VEC group, animals received VEC and, 30 min later, received VEC again, followed by a 20-min conditioning session; this procedure was repeated in the afternoon (5 h later). In the nicotine group, nicotine (0.5 mg/kg) was administered 30 min after VEC, followed immediately by a 20-min conditioning session; this procedure was repeated 5 h later. In the nicotine + ivermectin groups, nicotine (0.5 mg/kg) was injected 30 min after ivermectin administration (1 mg/kg or 5 mg/kg), followed immediately by 20 min of conditioning; the same dosing and timing were repeated in the afternoon session.

**Post-conditioning test (Day 6):** Mice were placed in the center of the apparatus with the doors open, allowing free access to both chambers for 15 min. No injections were administered.

**Investigation of the effect of ivermectin on extinction (Days 7–10):** VEC was administered to both the nicotine and VEC groups. The ivermectin groups received ivermectin (1 or 5 mg/kg) as appropriate. On days 1 and 4 (test days 7 and 10) of this period, animals were tested in the CPP apparatus for 15 min immediately following VEC and ivermectin injections, and the time spent in each chamber was recorded.

**Investigation of the effect of ivermectin on reinstatement (Day 11):** The nicotine group received VEC 30 min prior to nicotine administration. Immediately following nicotine injection, the animals were placed in the CPP apparatus for a 15-min test. The VEC group received VEC for both injections. The ivermectin groups received nicotine 30 min after ivermectin administration and were immediately tested in the apparatus for 15 min.

### Locomotor activity

After completing pre- and post-conditioning behavioral assessments, neurological deficits were evaluated sequentially using the Locomotor Activity Device and the rotarod apparatus. Mice were observed for 10 min in the Locomotor Activity Device, during which grooming, sniffing, head bobbing, and distance traveled were quantified.

### Rotarod test

The rotarod paradigm is a widely recognized experimental method for assessing motor coordination and postural control. It is commonly utilized to detect locomotor dysfunctions and balance disturbances resulting from exposure to addictive substances and various neurological disorders. The procedure involves placing an animal on a rotating rod, and the duration for which the animal maintains its balance while moving on the apparatus is recorded. In this study, assessments were conducted for 5 min following both acquisition and reinstatement sessions. Each subject participated in five trials: the first two served as adaptation and training, while the final three were designated for data analysis. A cut-off time of 300 s was established for each trial.

### Ethical statement

All experiments were conducted in compliance with NIH guidelines for the care and use of male Swiss albino mice (8 weeks old, 22–25 g). Ethical approval for the study was granted by the local animal experiments ethics committee (Decision number: 2020/08-09).

### Statistical analysis

The normality of the data distribution was assessed using the Kolmogorov–Smirnov test. Intergroup comparisons of normally distributed observations (*P* > 0.05) were conducted using one-way analysis of variance (ANOVA), while those of non-normally distributed observations (*P* < 0.05) utilized the nonparametric Kruskal–Wallis *H* test. For pairwise comparisons, parametric data were analyzed with the independent *t*-test, and nonparametric data were examined using the Mann–Whitney *U* test. The homogeneity of variances was assessed with Levene’s test. For groups with homogeneous variances, standard one-way ANOVA followed by Tukey’s post hoc test was applied for multiple comparisons. In instances where the assumption of homogeneity was not met, Welch’s ANOVA was used, followed by the Games–Howell post hoc test. The Benjamini–Hochberg false discovery rate (FDR) method was employed to adjust *P* values for multiple comparisons, applied separately within each predefined family of tests (e.g., acquisition/post-test; extinction day 1; extinction day 4; reinstatement). Adjusted *P* values in the tables reflect BH-FDR correction within the respective families. Although all animals were measured across experimental phases, individual micelles were not tracked, and samples were randomly selected for each measurement; consequently, repeated measures were not performed, and comparisons across time points were analyzed as independent observations. For each planned pairwise comparison, we report group means ± standard deviations (Mean ± SD), mean differences (I–J) with 95% confidence intervals, *P* values, BH-FDR adjusted *P* values, and ANOVA effect sizes (η^2^). Planned contrasts were conducted to compare specific group differences, with contrast estimates calculated for each relevant comparison. A priori sensitivity analysis using G*Power indicated that with four groups of six to eight animals each, the study was adequately powered (1-β ≈ 0.80) to detect large effect sizes (Cohen’s *f* ≈ 1.0, η^2^ ≈ 0.50) for the primary acquisition and reinstatement comparisons at α adjusted by the Benjamini–Hochberg procedure. These analyses were performed using SPSS, as detailed in the Supplemental data. All statistical analyses were carried out using SPSS software (version 21.0, IBM Corp., Armonk, NY, USA).

## Results

### Effects of treatment with ivermectin on the acquisition (development) of nicotine-induced CPP

**Figure 2.**
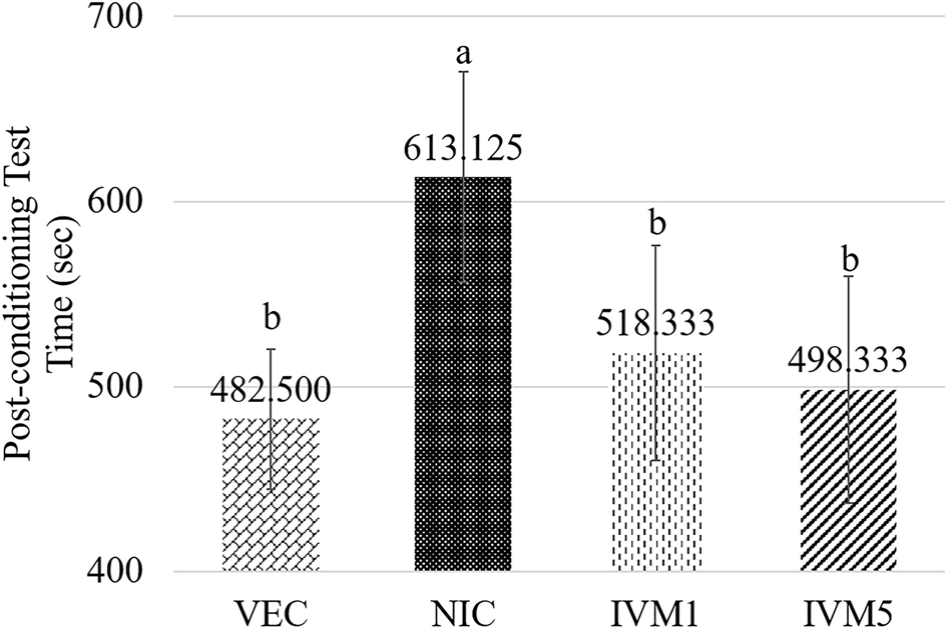
**Effects of ivermectin on nicotine-induced CPP acquisition.** During the post-conditioning test, NIC-treated mice spent significantly more time in the nicotine-paired chamber compared to VEC controls, confirming successful CPP induction. Both IVM 1 mg/kg and IVM 5 mg/kg groups showed a significant reduction in time spent in the conditioned chamber relative to the NIC group, indicating attenuation of nicotine-induced reward-like behavior. Data are presented as mean ± SD. a,b: Different letters indicate statistically significant differences among the means. Abbreviation: CPP: Conditioned place preference; VEC: Vehicle; NIC: Nicotine; IVM: Ivermectin; SEM: Standard error of the mean.

The effects of the experimental groups on post-conditioning test duration were assessed using one-way ANOVA, with data confirmed as normally distributed via the Kolmogorov–Smirnov test and homogeneity of variances validated by Levene’s test (*P* > 0.05). The analysis indicated a statistically significant difference among the groups during the post-conditioning test (η^2^ ═ 0.532, *P* < 0.001). Post hoc comparisons using Tukey’s multiple comparison test (Tukey’s HSD, α ═ 0.01) revealed that the NIC group (613.13 ± 56.83) spent significantly more time in the conditioned chamber than both the VEC group (482.5 ± 37.86; mean difference = 130.62 s, 95% CI [48.91, 212.34], adj. *P* ═ 0.007), the IVM 1 mg/kg group (518.33 ± 57.96; mean difference = 94.79 s, 95% CI [13.08, 176.50], adj. *P* ═ 0.038), and the IVM 5 mg/kg group (498.33 ± 61.21; mean difference = 114.79 s, 95% CI [33.08, 196.50], adj. *P* ═ 0.012) (Figure 2). A comparison between the NIC group and the control group showed a significantly longer test duration in the NIC group, confirming the successful establishment of the CPP model (*P* < 0.001). In contrast, both the IVM 1 mg/kg and 5 mg/kg groups exhibited a significant reduction in test duration compared to the NIC group, indicating a marked decrease in reward-like behavior. These findings suggest that both doses of IVM positively influenced nicotine dependence.

### Effects of ivermectin administration on the extinction of nicotine-induced CPP

The normality of the data distribution was assessed using the Kolmogorov–Smirnov test, which confirmed a normal distribution. Additionally, the homogeneity of variances was evaluated with Levene’s test, affirming that the assumption of homogeneity was met. The impact of the groups on the time spent in the drug-paired side (measured in seconds) was analyzed during the post-conditioning test, Extinction Test 1 (Ext Test 1), and Extinction Test 2 (Ext Test 2) using one-way ANOVA. The analysis revealed statistically significant differences between groups during the post-conditioning test and Ext Test 1 phases (η^2^ ═ 0.524, *P* < 0.01), while no significant differences were observed in Ext Test 2 (*P* ═ 0.22).

**Figure 3.**
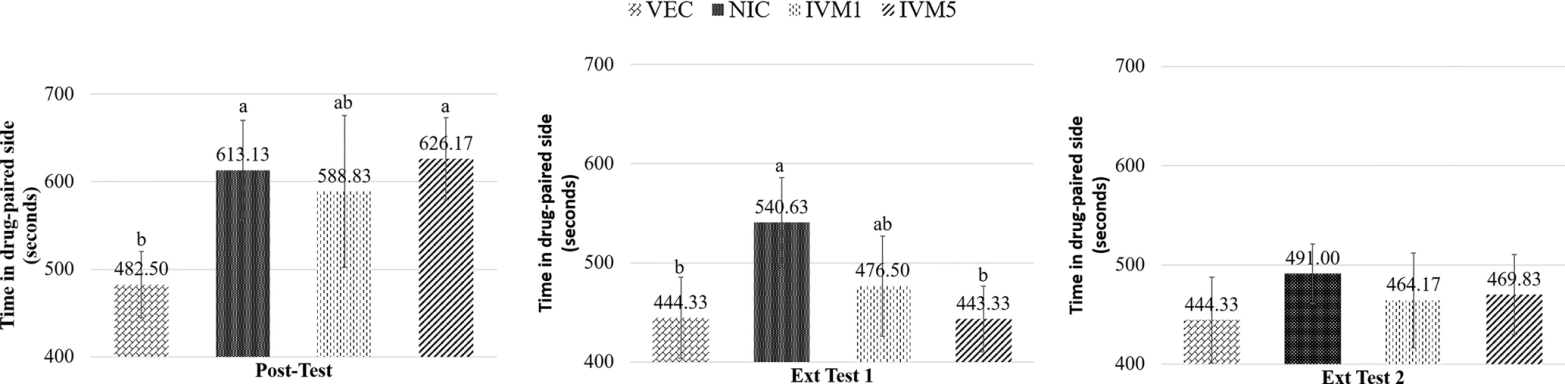
**Effects of ivermectin on the extinction of nicotine-induced conditioned place preference in mice.** The duration (in seconds) that mice spent in the nicotine-associated compartment was measured during the post-conditioning test across different groups: Nicotine only, vehicle (saline), and nicotine combined with ivermectin. During the extinction phase, animals in the ivermectin treatment groups (1 and 5 mg/kg, i.p.) received daily ivermectin injections, while saline was administered to both the control and nicotine-only groups. Extinction tests were carried out at one-day intervals (on days 7 and 10) and continued until the time spent in the nicotine-paired chamber became comparable to that of the control group. The results are expressed as mean ± SD, *n* ═ 6–8 mice per group. Abbreviations: NIC: Nicotine; VEC: Vehicle.

For the post-conditioning test, the normality of the data distribution was again evaluated using the Kolmogorov–Smirnov test, indicating a normal distribution. However, Levene’s test showed a violation of the homogeneity of variances assumption (*P* ═ 0.03). Consequently, group comparisons were conducted using Welch’s ANOVA, and multiple comparisons were performed with the Games–Howell post hoc test. Differences among groups in Extinction Test 1 were assessed using Tukey’s multiple comparison test. During Ext Test 1, the NIC group (540.63 ± 45.43 s) spent significantly more time in the drug-paired side compared to both the VEC group (444.33 ± 40.87 s; mean difference = 96.29 s, 95% CI [31.50, 161.08], adjusted *P* ═ 0.007) and the IVM 5 mg/kg group (443.33 ± 33.28 s; mean difference = 97.29 s, 95% CI [32.50, 162.08], adjusted *P* ═ 0.007). In contrast, the IVM 5 mg/kg group demonstrated a significantly reduced time (*P* < 0.01) spent in the drug-paired side compared to the NIC group, suggesting that high-dose ivermectin facilitated extinction (Figure 3). No significant difference (*P* > 0.05) was observed in the duration of Ext Test 1 for the IVM 1 mg/kg group. In Ext Test 2, no significant differences were detected among the groups, indicating that nicotine dependence had been effectively extinguished at this stage (*P* > 0.05).

### Effects of ivermectin administration on the reinstatement of nicotine-induced CPP

**Figure 4.**
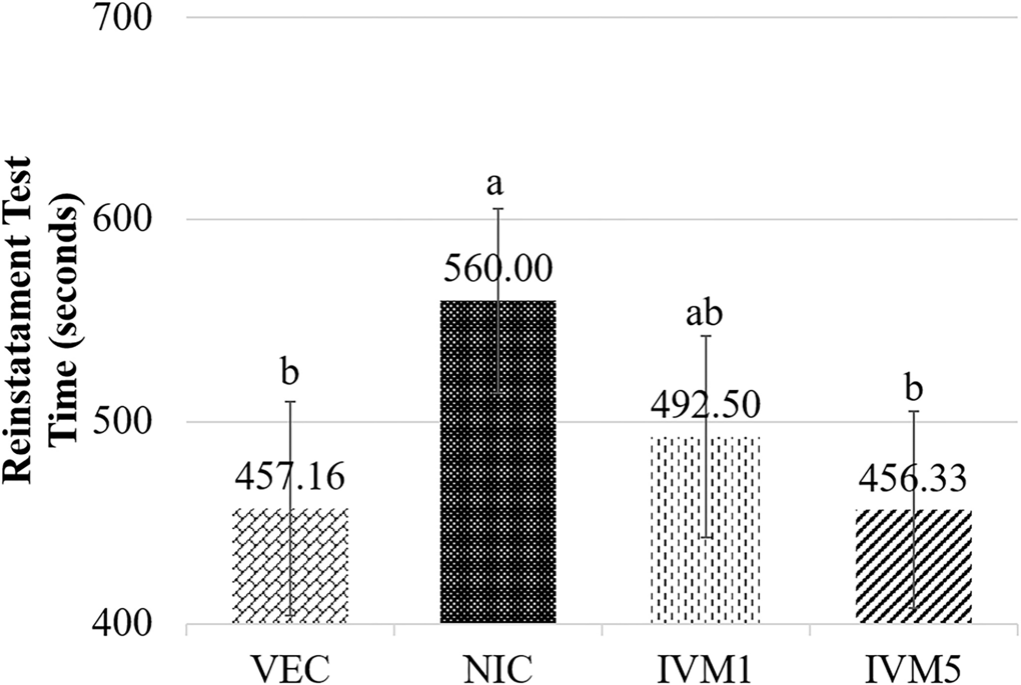
**Effects of ivermectin administration on the reinstatement of nicotine-induced conditioned place preference.** One day after extinction, and 30 min before nicotine (0.1 mg/kg, i.p.) was administered, saline was given to the nicotine group, and ivermectin was administered to the ivermectin (1 and 5 mg/kg, i.p.) groups. Physiological saline was administered to the control group. The results are expressed as mean ± SD, *n* ═ 6–8 mice per group. Abbreviations: NIC: Nicotine; VEC: Vehicle.

**Figure 5.**
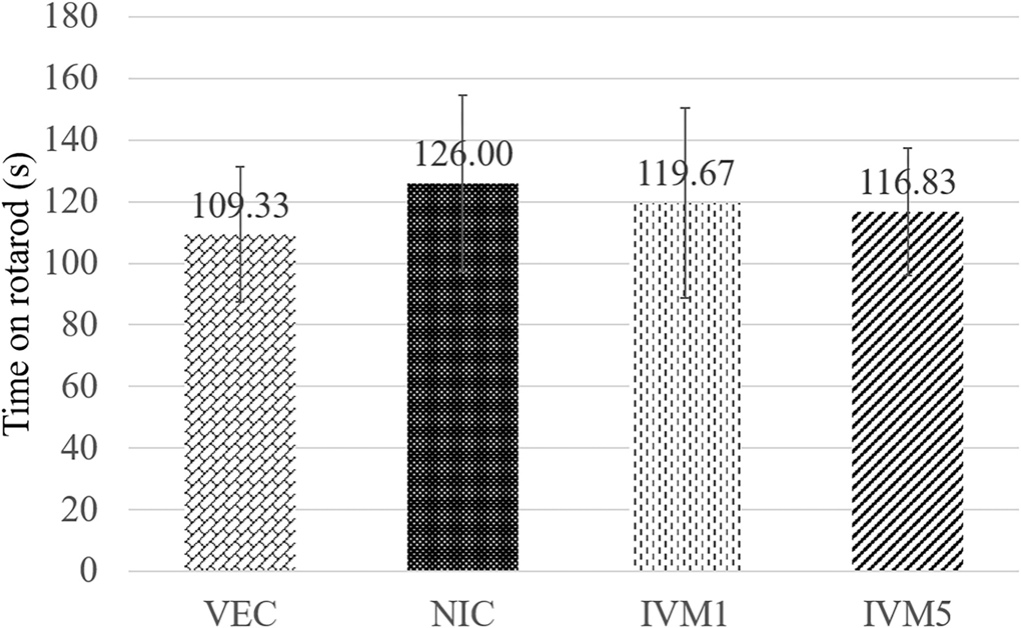
**Influence of ivermectin administration on performance in the rotarod assay.** The data were analyzed with one-way ANOVA. All measures shown as group means ± SD. *n* ═ 6–8 mice per group. Abbreviations: NIC: Nicotine; IVM: Ivermectin; VEC: Vehicle.

Reinstatement test durations (measured in seconds) were analyzed using one-way ANOVA, which revealed statistically significant differences among the groups (η^2^ ═ 0.492, *P* < 0.01). Levene’s test confirmed the homogeneity of variances (*P* ═ 0.856). Following this, Tukey’s multiple comparison test identified significant pairwise differences between the groups (see Figure 4). Independent samples *t*-tests were conducted to further evaluate group differences between reinstatement and pre-conditioning test values. This analysis indicated that, with the exception of the NIC group (560.00 ± 45.57), no statistically significant differences were found between the pre-conditioning and reinstatement values in the other groups. Notably, the NIC group spent significantly more time in the drug-paired compartment compared to the control (VEC) group (457.17 ± 52.95; mean difference = 102.83 s, 95% CI [29.24, 176.42], adjusted *P* ═ 0.012), indicating a reinstatement of nicotine-seeking behavior (*P* < 0.05). In contrast, the IVM 5 mg/kg group (456.33 ± 48.82; mean difference = 103.66 s, 95% CI [30.08, 177.26], adjusted *P* ═ 0.012) exhibited a significantly reduced time spent in the drug-paired compartment compared to the NIC group (*P* < 0.05). These findings suggest that IVM at a dosage of 5 mg/kg attenuates the reinstatement of nicotine dependence (see Figure 5).

### Impact of ivermectin on locomotor activity in nicotine-induced CPP

To assess the effects of the experimental groups on grooming, sniffing, head bobbing, and distance moved, data obtained from the post-conditioning and reinstatement tests were analyzed using Kolmogorov–Smirnov normality tests and Levene’s homogeneity tests. The normality analysis indicated that sniffing behavior during the post-conditioning test did not follow a normal distribution (*P* < 0.05), while all other measures adhered to normal distribution (*P* > 0.05). Consequently, the Kruskal–Wallis *H* test was employed for multiple comparisons of sniffing behavior, whereas one-way ANOVA was utilized for group comparisons of the other variables. Levene’s homogeneity test confirmed that all measures were homogeneously distributed (*P* > 0.05). As shown in Table 1, no statistically significant differences were identified among the groups for any of the assessed variables (*P* > 0.05).

**Table 1:** Effects of nicotine on the locomotor activity of mice receiving it alone or in combination with ivermectin (Mean ± SD)

**Parameter**	**VEC**	**NIC**	**IVM 1**	**IVM 5**	***P***
*n*	6	8	6	6	
*Post-conditioning phase*					
Grooming (s)	2.00 ± 1.41	3.38 ± 1.69	2.50 ± 1.05	2.00 ± 1.41	0.250
Sniffing (s)	2.17 ± 1.72	2.38 ± 1.60	2.83 ± 1.33	2.83 ± 1.17	0.813
Distance moved (cm)	3261 ± 734	4127 ± 1166	3500 ± 1124	3688 ± 994	0.462
*Reinstatement test*					
Grooming (s)	2.17 ± 1.47	3.00 ± 2.20	2.83 ± 2.48	2.66 ± 1.97	0.898
Sniffing (s)	2.33 ± 2.07	3.38 ± 1.85	2.50 ± 1.38	2.33 ± 2.07	0.666
Distance moved (cm)	3199 ± 510	3855 ± 1447	3912 ± 328	3550 ± 634	0.505

Abbreviations: VEC: Vehicle (control); NIC: Nicotine; IVM: Ivermectin; IVM 1: Ivermectin (1 mg/kg); IVM 5: Ivermectin (5 mg/kg); SD: Standard deviation.

The analysis of behavioral parameters (grooming, sniffing, head bobbing, distance moved) in the post-conditioning test yielded no significant results. Additionally, head bobbing behavior was not observed in all groups, precluding any further analysis of this parameter.

### Effect of ivermectin on motor coordination in nicotine-induced CPP

One-way ANOVA analysis indicated that neither nicotine alone nor its combination with ivermectin significantly impacted motor coordination (*P* > 0.05) (Figure 5).

## Discussion

This study investigates the effects of ivermectin on the rewarding properties of nicotine, as measured by various stages of CPP. Our findings suggest that ivermectin, an FDA-approved antiparasitic agent with positive allosteric modulator activity at GABA-A and P2X4 receptors, shows promising potential in addressing addiction. Among the pharmacological treatments available to alleviate addiction symptoms, few have the capability to reduce drug acquisition, and even fewer are effective in preventing reinstatement [36, 37].

While CPP does not directly assess the stages of nicotine addiction, it mimics the behavior of human smokers conditioned to smoke in environments previously associated with reward [38]. Consistent with existing literature, mice administered nicotine (0.5 mg/kg, i.p.) exhibited CPP by spending significantly more time in the nicotine-paired chamber compared to the saline-paired chamber [29, 37, 39]. In line with previous findings, no statistically significant differences were observed in locomotor activity or rotarod tests [37, 40]. The doses of ivermectin and nicotine used in this study were selected based on effective doses identified in prior research [18, 20, 41].

Our study, consistent with other CPP-related research, found that ivermectin effectively facilitated extinction [29, 42, 43]. This study is the first to present data indicating that ivermectin reduces psychological dependence on nicotine and its reinstatement. Extinction and reinstatement, integral components of drug and substance dependence, pose significant challenges in addiction treatment [35, 44]. These issues can exacerbate the burden on healthcare services, leading to increased utilization, including emergency care [44].

While only one study has specifically examined avermectin in tobacco-related models, Chen et al. [45] demonstrated that the deletion of sphingosine kinase 2 mitigated cigarette smoke-induced COPD-like symptoms in mice, indicating a potential link between avermectin-related mechanisms and tobacco-induced pathology. Although this research focused on cigarette smoke exposure and lung outcomes rather than behavioral nicotine addiction, it provides essential context regarding the role of avermectin-related pathways in tobacco-related diseases. Our findings build on this work by extending the investigation from pulmonary outcomes to behavioral addiction processes, thereby enhancing the understanding of ivermectin’s potential in nicotine-related health contexts.

Nicotine demonstrates rewarding effects by inducing dopamine release, primarily through nicotinic acetylcholine receptors. Ivermectin has been identified as a positive allosteric modulator of α7 nicotinic acetylcholine receptors (α7nAChR) and is known to induce receptor desensitization, suggesting its potential to modulate nicotine’s rewarding effects directly [20, 46, 47]. Additionally, ivermectin may diminish nicotine-induced CPP through various pharmacological mechanisms. Given that P2X4 receptors (P2X4Rs) positively modulate GABA_A receptors, glycine receptors, and nicotinic acetylcholine receptors—key targets in addiction—an interaction between P2X4Rs and these ionotropic receptors may underlie the *in vivo* pharmacological effects of ivermectin [20]. As a positive allosteric modulator of P2X4Rs, which are crucial in purinergic signaling in brain regions linked to reward and addiction, ivermectin may weaken the reward signal by altering nicotine’s effects on its receptors via this pathway [23, 24].

Alterations in P2X4R function have been associated with dopamine depletion [48]. Dopamine, a neurotransmitter traditionally linked to the reinforcing effects of addictive substances, plays a significant role in triggering neurobiological changes associated with addiction [48]. Recent studies indicate that P2X4Rs may influence the reward circuit by modulating dopamine or glutamate release within the ventral tegmental area and the nucleus accumbens. Furthermore, ivermectin has been shown to increase the firing rate of striatal cholinergic interneurons, suggesting that it elevates dopamine levels not primarily through changes in vesicular content but through increased terminal excitability [16]. Thus, it is hypothesized that ivermectin may impact addiction by enhancing cholinergic activity at dopamine terminals, facilitating dopamine release in the striatum.

GABA, synthesized from glutamate in brain cells, primarily functions as an inhibitory neurotransmitter. Nicotine modifies GABA activity in the brain through various mechanisms [41]. GABA agonists have been shown to reduce both nicotine addiction and reinstatement while accelerating the extinction of nicotine-CPP [41, 49]. Recent studies have also reported that ivermectin stimulates GABA activity [18, 50, 51]. This finding aligns with previous research indicating that GABA receptor agonists typically produce inhibitory effects on CPP.

Contemporary pharmacological strategies for addressing substance and drug dependence focus on modulating or inhibiting the effects of drugs at their sites of action, targeting the critical phases of extinction, acquisition, and reinstatement [35]. However, few pharmacological treatments effectively reduce drug acquisition, and even fewer succeed in preventing reinstatement [35]. Numerous experimental and clinical studies have confirmed that re-exposure to a drug (priming) is a critical event correlated with drug-seeking behavior in both humans and animals [52].

Notably, ivermectin not only mitigates extinction syndrome but also prevents the development of reinstatement, a particularly resistant stage of addiction. In our study, even a low priming dose of nicotine (0.1 mg/kg) administered to previously exposed individuals did not result in the reappearance of nicotine-induced CPP when ivermectin was present. Relapse is a hallmark of chronic addiction and a primary factor limiting the long-term efficacy of existing pharmacotherapies [14]. For instance, while treatments such as varenicline and bupropion initially enhance quit rates, relapse rates exceed 60% within one year [13].

These findings suggest that ivermectin may not only prevent the onset of addiction but also play a crucial role in relapse prevention, particularly against stress-induced or drug-reexposure triggers, addressing a significant gap in addiction treatment [53]. The multifaceted effects of ivermectin underscore the urgent need for more comprehensive and innovative pharmacological strategies for managing nicotine addiction.

Several limitations must be acknowledged in this study. First, the antiparasitic agent ivermectin typically exhibits limited brain penetration in vertebrates due to effective efflux at the blood–brain barrier mediated by P-glycoprotein, which is encoded by the multidrug resistance gene. Second, this study focused exclusively on male mice, despite evidence that gender differences significantly influence all stages of nicotine addiction, from initiation to withdrawal and relapse. Previous research suggests that females may be more sensitive to the rewarding effects of nicotine and develop dependence at lower doses, with a more rapid progression than males [54]. Thus, the exclusion of females limits the generalizability of our findings. Third, as rodents are predominantly active during the dark phase, the timing of our behavioral testing, conducted during daylight hours, may have influenced the outcomes. Future CPP studies with ivermectin could benefit from including both sexes and conducting experiments during the animals’ active (dark or twilight) phase to better account for natural circadian and sex-dependent variability.

Another limitation is the absence of a DMSO vehicle control group. However, previous behavioral studies with experimental designs similar to ours have shown that DMSO administration alone does not significantly alter locomotor activity or performance on the rotarod test [55–62]. Likewise, in CPP paradigms comparable to the current investigation, DMSO has not been shown to exert a statistically significant effect on CPP outcomes [58–62]. Nonetheless, the lack of a dedicated DMSO control group may complicate the interpretation of our findings. Therefore, future studies should include a DMSO vehicle group to strengthen the validity and interpretability of behavioral outcomes.

Additionally, further investigation is needed into the dose-response relationship, safety profile, and potential interactions with other psychoactive substances. While this study’s findings are based on existing biological evidence regarding the effects of ivermectin on nicotine dependence, direct clinical studies involving human subjects have yet to be conducted. Although the synaptic effects and pharmacokinetic profile of ivermectin are well characterized, its long-term effects on addictive behaviors remain inadequately understood.

### Future directions

Future research should incorporate controlled experimental designs in both animal models and human populations to assess the therapeutic effects of ivermectin on nicotine addiction. Specifically, more comprehensive analyses are needed using models that simulate various stages of addictive behavior, including CPP, self-administration, withdrawal, and relapse. Clinically, the applicability, efficacy, and tolerability of ivermectin in emergency department settings should be rigorously evaluated and substantiated through comparative studies with existing pharmacotherapies.

## Conclusion

In this study, we demonstrated that ivermectin significantly modulates nicotine-induced CPP in mice. High doses of ivermectin attenuated nicotine-related reward behavior, accelerated extinction processes, and effectively blocked reinstatement. These findings indicate that ivermectin influences reward circuitry through multiple receptor pathways, highlighting its translational relevance for nicotine dependence. Notably, ivermectin’s ability to suppress reinstatement—a critical factor in relapse—underscores its potential clinical utility. However, further mechanistic studies, dose–response analyses, and translational investigations are necessary to delineate its therapeutic profile. Collectively, our results advocate for ivermectin as a promising candidate for drug repurposing in addiction medicine, particularly in relapse prevention strategies.

## Supplemental data

Supplemental data are available at the following link: https://www.bjbms.org/ojs/index.php/bjbms/article/view/13026/4012.

## Data Availability

The data used in the current study have not been deposited in a public repository. However, the datasets supporting the findings of this study are available from the corresponding author [MED] upon reasonable request.
